# Children With Conduct Problems and Co-occurring ADHD: Behavioral Improvements Following Parent Management Training

**DOI:** 10.1080/07317107.2015.1000227

**Published:** 2015-03-09

**Authors:** Gunnar Bjørnebekk, John Kjøbli, Terje Ogden

**Affiliations:** ^a^ Norwegian Center for Child Behavioral Development, University of Oslo, Oslo, Norway

**Keywords:** ADHD, conduct problems, outcome predictions, parent management training

## Abstract

To scale up evidence-based treatment of conduct problems, parent management training, Oregon model (PMTO) has been disseminated throughout Norway. This study examined whether Attention Deficit Hyperactivity Disorder (ADHD) predicted the outcomes of PMTO. Of 253 children and families, 97 were reported to have an ADHD diagnosis. Although different at intake, the groups with and without ADHD had close to an equal change in behavioral status following treatment. Maternal depression and family income predicted the combined group's behavior following PMTO. The study indicates that reductions in conduct problems following PMTO are of the same magnitude in children with or without ADHD. However, some characteristics may differentially predict outcomes for children with combined problems.

Children with conduct problems (CP) and co-occurring Attention Deficit Hyperactivity Disorder (ADHD) problems have been identified as being at high risk of lifelong trajectories of antisocial and delinquent behavior (Gresham, Lane, & Lambros, 2000). High levels of childhood inattention and impulsivity place multiple demands on parents and increase the likelihood that they will respond with harsh discipline, less positive parenting practice, inappropriate withdrawal from the child, or inconsistent parenting (Deault, 2010; Johnston & Jassy, 2007). The children's impulsive response style interacts with the tendency to engage in various forms of acting-out behavior (Manuzza, Klein, Abikoff, & Moulton, 2004), and is linked to increased risk of negative outcomes in adulthood—such as substance abuse, accidents, and dependence on welfare (Walker, Lahey, Hynd, & Frame, 1987). These findings underline the importance of early intervention in order to prevent further problem development in children with co-occurring symptoms of CP and ADHD.

Meta-analyses have documented that parent management training (PMT) is an effective treatment for children with conduct problems (Lundahl, Risser & Lovejoy, 2006) and for children with ADHD (Lee, Niew, Yang, Chen & Lin, 2012; Fabiano et al., 2009). However, only a limited number of intervention studies exist that contribute to our understanding of how attention and hyperactivity problems influence treatment outcomes for children with CP.

## INTERVENTIONS TARGETING CHILDREN WITH CP AND ADHD

ADHD and CP tend to co-occur. In a recent population-based birth cohort study, 22.5% of the children diagnosed with ADHD also met diagnostic criteria for Conduct Disorder/Oppositional Defiant Disorder (CD/ODD; Yoshimasu et al., 2012). Moreover, many children referred to treatment because of conduct problems also have co-occurring ADHD (Scott, Knapp, Henderson, & Maughan, 2001). It has been claimed that children with ADHD and ODD might represent a distinctive pattern of dysfunction that is different from each of the diagnoses individually (Hinshaw, 1994), and that they might suffer from a particularly refractory condition that renders them less responsive to interventions (Lynam, 1998). Reviews of the literature indicate that these children may require more intensive services (Gresham et al., 2000; Ollendick, Jarrett, Grills-Taquechel, Hovey, & Wolff, 2008). In a meta-analysis reviewed 16 studies of parent-involved treatment for children with ADHD, the effects on ADHD-symptoms and behavioral problems were relatively minor (.40 and .36; Corcoran & Dattalo, 2006). Based on their results compared to the effects for medication on ADHD symptoms and aggression, Corcoran and Dattalo (2006) argued that children with combined problems are better served by other interventions. Moreover, the results from Lee et al.'s (2012) meta-analyses showed that children with ADHD and comorbid CP benefited less from parent training than children with ADHD only.

These findings and conclusions have been challenged by some recent studies, a meta-analysis and reviews that indicate that ADHD does not predict negative outcomes for children with CP. Rather than requiring more intensive services and being less responsive to interventions, some findings suggest that comorbid children sometimes respond better to treatment than children with only CP (Hartman, Stage, & Webster-Stratton, 2003; Ollendick et al., 2008). Beauchaine, Webster-Stratton, and Reid (2005) combined data from six randomized controlled trials (RCTs) of the Incredible Years parent training program and found that attention problems combined with CD neither moderated nor predicted outcomes of parent training when delivered in a group-based format. Moreover, in Fabiano et al.'s (2009) meta-analysis of behavioral treatment for ADHD, child comorbidity was not associated with study effect size.

Some variables have been found to predict treatment outcomes in separate studies of ADHD and CP, and also in a few combined studies. Maternal depression is assumed to have a negative impact on parent training outcomes for children with conduct problems (Reyno & McGrath, 2006) and children with ADHD: In the Multimodal Treatment Study of Children with ADHD (MTA), parental depressive symptoms were associated with decreased response rates across treatment modalities (Owens et al., 2003). On the other hand, Zachor, Hodgens, and Patterson (2009) found no relationship between maternal depression and decreased effectiveness of parent training in families of children with ADHD. Low scores on socioeconomic status (SES) variables such as low income and education, and also single-parent status have been associated with a poorer behavioral treatment response in parent training of children with CP (Reyno & McGrath, 2006; Shelleby & Kolko, 2013). Corcoran and Dattalo's (2006) meta-analysis indicated also that children with ADHD in single-parent homes did not do as well in parent training as those who were from two-parent homes. As the above findings suggest, findings are mixed, and whether SES-variables, maternal depression, or single parent-status differentially predict outcomes for children with CP plus ADHD compared to CP only is uncertain.

Research has also suggested that there might be some differences between ADHD plus CP groups and CP-only groups at intake to treatment. In a study by Hartman et al. (2003), where the authors examined whether risk factors influenced parent training outcomes, children with combined CP and attention-related problems scored significantly higher on externalized behavior problems than children with CP-only. Findings showed, however, that combined attentional problems and CP did not negatively affect the outcomes of parent training. Moreover, Gittelman, Mannuzza, Shenker, and Bonagura (1985) found, in a longitudinal study of adolescents who had been diagnosed hyperactive in childhood, that ADHD plus CD was related to a higher frequency of conflicts with teachers and more fighting and stealing than a CD-only group. Also, findings have showed children with ADHD plus CP to be significantly delayed in their play and social skills (Webster-Stratton & Lindsay, 1999) and to have lower scores on oral intelligence (Moffitt, 1990) than CP-only groups.

In Norway, a policy decision to scale up the use of evidence-based intervention to reduce child conduct problems has resulted in a nationwide dissemination of parent management training (the Oregon model: PMTO; Ogden, Forgatch, Askeland, Patterson, & Bullock, 2005) as part of the child and adolescent psychiatric and child welfare services. A randomized controlled trial (RCT) conducted in Norway showed that PMTO was effective in reducing parent-reported child externalizing problems, improving teacher-reported social competence, and enhancing parental discipline compared to regular services at postassessment (Ogden & Amlund-Hagen, 2008) and that the behavioral outcomes were sustainable 1 year later (Amlund-Hagen, Ogden, & Bjørnebekk, 2011).

To the authors’ knowledge, no studies have examined how attention and hyperactivity problems influence treatment outcomes for children with CP in individually administered PMTO. Taken together with the mixed empirical evidence of the impact of ADHD on outcomes of parent training programs for children with CP, the present study was set out to examine whether children with CP, as compared with children with combined CP and ADHD, did differ in social background at intake to treatment and whether their amount of behavioral change during PMTO would be comparable. Moreover, as results from earlier studies have suggested that children with combined CP and ADHD score higher on the problem scales (attention, social, and externalizing) and lower on social skills and academic competence at intake, we wanted to examine whether this was the case in the present study. We also examined whether individual differences in behavioral change in the combined CP and ADHD group would be predicted by variables previously demonstrated to moderate outcomes in the treatment of children with CP or ADHD (i.e., maternal depression: family income level, and single parent-status). Specifically, the research questions were:

1. Do children with combined CP and ADHD differ from children with CP only at intake to treatment, and do the two groups differ in terms of behavioral change following treatment?
2. Is the influence of family income level, single parent-status, and maternal anxiety/depression on the children's behavioral change different in the combined CP and ADHD group and the group with CP only?

## METHOD

### Participants

During the recruitment period between January 2001 and April 2005, 331 children with conduct problems and their families received PMTO via Child and Adolescent Mental Health agencies and Child Welfare Services (of these, 58 had participated in an RCT; Ogden & Amlund-Hagen, 2008). To match the regular procedures for referral or intake to child and family services, no formal screening procedures were used as part of this study; rather, the intervention was offered based on the practitioners’ clinical judgments. Children were not included in the study if they (a) were diagnosed with autism, (b) had been exposed to documented sexual assault, (c) had intellectual disabilities, (d) had parents with serious mental health problems or had intellectual disabilities, or (e) diagnostic information was unavailable. Among the referred families, 253 were included in the present study based on parent, teacher, and/or PMTO therapist reports of child ADHD diagnosis. Seventy-eight families were excluded in the present study because of missing information about if the child had been diagnosed or not. Children's previous diagnoses of ADHD were determined by the Child and Adolescent Mental Health agencies in Norway with the use of standardized assessment procedures. Although the reports indicated whether children had been diagnosed based on standardized assessments conducted by the Child and Adolescent Mental Health agencies in Norway, these diagnoses were not verified by independent diagnostic assessments at intake to the treatment. A total of 97 (38.3%) were reported to have ADHD.

Children under the age of 12 and their parents (or guardians) could be referred for treatment (*M* = 8.69, *SD* = 2.14), and of these 236 (73.1%) were boys. The average gross annual family income was NOK 416.558 (*SD* = 222.069), approximately US$68,625 (*SD* = $36.58), which in Norway represents a lower to middle income level. The average age of the reporting parent was 37.67 years (*SD* = 6.3). Thirty-five percent were single parents, 30% had a college or higher university degree, 52% had finished high school, and 18% had completed junior high or elementary school. Most of the parents (94% of *n* = 266) were of Norwegian ethnic background. Children could be referred irrespective of being medicated or not, but on condition that no changes could occur during treatment. Children who were on medication at intake were asked to continue this during treatment, and children who were not on medication were asked not to start during the trial.

### Procedures

Two-hour assessments were conducted before (pre) and after (post) PMTO treatment. Eligible families were informed about the study and signed written consent forms. The parent who reported to have spent the most time with the child rated the child's behavior. If parents agreed, then children's teachers were asked to fill out questionnaires about the child's behavior. Parents received NOK 300 (about US$50) and teachers received NOK 100 (about US$17) as compensation for their participation.

### Measures

*The Child Behavior Checklist* (CBCL) and *Teacher Report Form* (TRF; Achenbach, 1991) have been validated and standardized in Norwegian studies (Lurie, 2006; Nøvik, 1999). The externalizing (e.g., “gets in many fights”) and internalizing (e.g., “would rather be alone than with others”) composite, the attention (e.g., “can't sit still, restless, or hyperactive”), social (“prefers being with younger kids”), and the anxiety/depression (e.g., “cries a lot”) problems subscales were used in the present study. Items were rated on a 3-point Likert scale (0 = *never/seldom*, 1 = *sometimes*, 2 = *often/always*), in which higher scores represent greater symptomatology. Alpha coefficients at preassessment and postassessment were found to be within an acceptable range (from .73 to .96). Of the 253 included at preassessment, 19% of CBCL and 22% of the TRF assessments were missing at postassessment.

*The Social Skills Rating System* (SSRS; Gresham & Elliott, 1990) is a standardized, multirater instrument that assesses social skills in children and youth. The SSRS has been used with diverse samples, and its validity with Norwegian children was examined specifically in a study showing that scores on the SSRS covaried with teacher ratings of problem behavior, academic competence, and sociometric nominations by peers (Ogden, 2003). It has both a parent and a teacher version. The parent scale has 38 items (e.g., “receives criticism well”) and the teacher scale has 30 items (e.g., “invites others to join activities”). Pre- and post-Cronbach's alphas on the parent instrument were .87 and .89, and the pre-alphas and post-alphas on the teacher instrument were .86 and .86. At postassessment, ratings from 13% of the parents and 24% of the teachers were missing.

*Academic competence* was measured by teacher ratings on the 9-item scale of the SSRS (Gresham & Elliott, 1990). Items were rated on a 5-point scale according to the teachers’ rank order of the student in reading and mathematics performance, motivation, parental support, and general cognitive functioning (1 = *lowest 10%*, 2 = *next lowest 20%*, 3 = *middle 40%*, 4 = *next highest 20%*, and 5 = *highest 10%*; α = .87 at preassessment and α = .88 at postassessment). At postassessment, scores were missing for 10% of the 202 school-aged children in the study.

*The Parent Daily Report* (PDR) has shown to be a reliable and valid measure of conduct problems (Patterson, Chamberlain, & Reid, 1982) and support for its validity has been demonstrated by its association with independent observers’ accounts of child problem behaviors (e.g., Chamberlain & Reid, 1987). The PDR has formerly been adapted by a team of experts from the Oregon Social Learning Center and translated in order to be used in the Norwegian effectiveness study (Ogden & Amlund-Hagen, 2008). The participating parents were phoned on 3 successive days before and after treatment, and answered “yes” or “no” to whether specific child behaviors had occurred during the last 24 hours (item example: “hits other people”). The sum-score for each successive day was calculated as a total score for all 3 days. Reliability coefficients were acceptable, with alphas of .75 on and .78 at postassessment. Of the 253 families included at preassessment, 18% of the PDR reports were missing at postassessment.

*Maternal mental distress* (i.e., anxiety and depression) were measured by the Symptom Checklist-5 (SCL-5) which is a five-item scale based on SCL-25 has been proven valid and reliable in earlier Norwegian studies (Tambs & Moum, 1993), and has formerly been used in Norwegian clinical trials (Kjøbli & Bjørnebekk, 2013) and treatment studies (Kjøbli, Nærde, Bjørnebekk, & Askeland, 2013). Respondents indicated if he/she was bothered or distressed at all, was a little bit, quite much, or very much bothered by: (a) feeling fearful, (b) nervousness or shakiness inside, (c) feeling hopeless about the future, (d) worrying too much about things, and (e) feeling blue (α = .89 at preassessment and α = .89 at postassessment). Scores were missing for 18% of the cases at postassessment.

### Treatment—Parent Management Training

The treatment is principle-based and conducted according the PMTO manual or handbook (Askeland, Christiansen, & Solholm, 2005). During weekly sessions, parents met with a therapist to learn functional strategies through (a) increased encouragement of prosocial behavior (e.g., compliance and cooperation); and (b) contingent use of mild negative consequences for negative behavior, like the removal of privileges (response cost) or with the use of time-out. The families were treated individually and intervention was flexible enough to meet the individual needs of each family. Parents were the primary intervention targets as they were considered the agents of change in child outcomes. The therapy identifies and builds on strengths already present in the family. The positive parenting dimensions were introduced and practiced before the focus turned to issues of supervision, limit setting, and implementing negative contingencies. Each treatment session had a specific agenda and a typical PMTO session included: (a) greetings; (b) debrief of the home practice assignment; (c) reviews, troubleshooting, and brainstorming of previous materials; (d) introducing new skills; (e) role play and exercises; (f) addressing individual family needs; and (g) home practice assignment. Between sessions, parents were followed up with a midweek telephone call. PMTO typically requires 20–50 sessions in order to reach treatment goals, and families in this study received on average 24.4 hours in therapy (*M*_ADHD_ = 24 versus *M*_not ADHD_ = 24.6, *t*(1, 214) = 0.24, *p* = .65). All families who started PMTO-treatment were asked to participate in the data assessments, regardless of how many sessions of treatment they received.

The aim of PMTO is to enhance five central parenting skills: skill encouragement, monitoring/supervision, problem solving, positive involvement, and limit setting/discipline. *Skill encouragement* promotes competence through positive contingencies (Forgatch & DeGarmo, 1999). *Monitoring* protects youngsters from involvement in risky activities and it reflects parental tracking of children's whereabouts (Patterson & Forgatch, 2005). *Problem solving* helps family members negotiate disagreements, establish house rules, and specify consequences for following or violating rules (Patterson & Forgatch, 2005). *Positive involvement* reflects how parents demonstrate interest in, attention to, and care for their child (Forgatch & DeGarmo, 1999). Effective *discipline and limit setting* discourage deviant behavior through the appropriate and contingent use of mild sanctions (Patterson, 1986) and they provide the child with clear boundaries for acceptable behavior.

A treatment manual written by one of the program developers (Forgatch, 1994) was translated and adapted to Norwegian conditions and additional materials such as family check lists and reward cards were developed by the national PMTO implementation team (Askeland et al., 2005). PMTO candidates underwent 18 months of training and had to complete 3–5 full-scale PMTO therapies during their training periods. The candidates also attended regular booster sessions and met with their supervisors discussing their therapies, treatment obstacles, and clinical outcomes. Therapists were certified based on PMTO specialists’ assessments of video-recorded therapy sessions. The comprehensive training, certification, and ongoing supervision of the PMTO therapists helped secure treatment fidelity.

## RESULTS

### Analytic Procedures

#### Missing Data and Outliers

Missing data were replaced at the item level using the expectation maximization (EM) procedure (Tabachnik & Fidell, 2001). Imputation was performed on missing items only, thereby leaving out cases in which entire scales were missing. A missing completely at random (MCAR) test was carried out for each EM, and if this test failed, then regression analysis was used to impute missing values. Outliers were identified and inspected both at preassessment and postassessment, and the 5% trimmed mean was compared with the original mean. The outliers had little impact on the original mean and were therefore not modified. All scales were found to be within an acceptable range of skewness and kurtosis (+/−2), and consequently, no transformations of variables were conducted.

#### Analyses of Baseline Differences

One-way ANOVAs were carried out to examine group differences at baseline. Raw scores of the CBCL and TRF externalizing and internalizing behavior were also transformed into *T*-scores, based on gender-specific standardizations of the measures in Norwegian samples (Lurie, 2006; Nøvik, 1999). Scores at or above the 90th percentile (64 points) were considered to be in the clinical, as opposed to the normal, range (Achenbach, 1991). *T*-scores were reported because of their clinical relevance. Raw scores were used in the primary analyses.

#### Main Analyses

ANCOVAs were conducted to examine how ADHD predicted the outcome variables. In order to control for the influence that prescores, gender, and type of treatment organization had on postscores, these variables were included as covariates in the analyses. A series of hierarchical multiple regressions were carried out to examine the moderators of interest. In computing the interaction terms, consistent with Aiken and West (1991), the components were centered to minimize multicollinearity among the main effect and interaction term. Prescores of the outcome variable, the potential moderator, and ADHD were entered in Step 1. The two-way interaction terms were entered in Step 2. Significant interaction effects were interpreted by generating predicted values from the regression equations using the dummy codes for the categorical variable (*ADHD* = 0 and *not ADHD* = 1, respectively) and representative high and low scores (1 standard deviation above and below the mean, respectively) for the continuous variables. To verify the statistical significance of the interactions, simple slope tests were conducted (Preacher, Curran, & Bauer, 2006).

#### Differences at Intake to Treatment

At intake, the parent reports demonstrated that children with an ADHD diagnosis scored significantly higher on attention problems, *F*(1, 249) = 20.15, *p* < .001; externalizing problems, *F*(1, 249) = 7.42, *p* < .001; and social problems, *F*(1, 249) = 11.35, *p* < .001, than those without. The teacher reports (TRF) showed that children with ADHD scored significantly higher on attention problems, *F*(1, 223) = 15.61, *p* < .001; externalizing problems, *F*(1, 223) = 5.36, *p* < .05; and social problems, *F*(1, 223) = 7.11, *p* < .01; but also lower on social skills, *F*(1, 222) = 9.36, *p* < .01; and academic competence, *F*(1, 202) = 5.51, *p* < .05. This indicates that the group reported to have the ADHD diagnosis scored significantly higher on both parent and teacher ratings of attention problems, indicating the validity of the parent and/or therapist reports of ADHD. Furthermore, based on normative scores adjusted for age and gender, the mean *T*-score was 84.36 for the group with ADHD diagnosis on the Child Behavior Checklist (CBCL) attention problem scale. Thus, the average child in the group reported to have an ADHD diagnosis scored about 3.5 standard deviations above the normative mean for Norwegian children.

#### Children With and Without Reports of ADHD Diagnosis

The children reported to have a diagnosis of ADHD were compared with those who were excluded from analyses because of missing information about ADHD. At pretreatment, parent age was significantly lower in the included group (i.e., those with information about ADHD), *t*(1, 223) = −2.52, *p* < .01. Furthermore, children in the included group scored higher on CBCL attention problems, *t*(1, 315) = 2.41, *p* < .02; and TRF attention problems, *t*(1, 279) = 2.85, *p* < .005. In order to probe this finding, we ran an ANOVA to test for differences between the excluded group, the ADHD group, and the group without ADHD. The ANOVAs (using Bonferroni post hoc test criteria) revealed a significant difference between the ADHD group and the excluded group on both measures, while differences were nonsignificant between the excluded group and the group without ADHD. This indicates that most of the excluded cases did not qualify for a diagnosis of ADHD.

### Attrition

Of all the families, 232 (92%) completed one or more of the instruments at postassessment. At pretreatment, there were no significant differences between the attrition group and the completers on background and child behavior variables.

### Baseline Comparisons

When *children with and without diagnosed ADHD* were compared on demographic characteristics, there were no significant baseline differences between the two groups on child age, family income, parent age and education, and number of siblings. In addition, single-parent status, child's ethnicity, and receiving welfare did not differentiate the two groups. Significantly more children diagnosed with ADHD were recruited from Child and Adolescent Mental Health services (*N* = 61) than from Child Welfare Services (*N* = 36; χ^2^ = 18.39, *p* < .001) and the majority were boys. Gender and type of organization were therefore included as covariates in the main analyses.

### Main Effects of ADHD on Behavioral Outcomes

In all models, prescores for the outcome, the type of treatment organization, and child gender were controlled. Overall, the variable *ADHD or not* predicted only 1of 14 outcomes in this study (marginally significant); children with an ADHD diagnosis were rated by their parents to score lower on social skills, *F*(1, 214) = 2.69, *p* < .10, after PMTO treatment (Table 1). Further, at the end of treatment, the group with both ADHD and CP still scored higher on both teacher, *F*(1, 195) = 3.89, *p* < .05, and parent reported, *F*(1, 203) = 9.33, *p* < .004, attention problems; and on teacher, *F*(1, 191) = 5.02, *p* < .04, and parent reported, *F*(1, 219) = 4.17, *p* < .05, social competence. However, on parent- and teacher-reported externalizing and social problems, and on academic competence, the groups did not differ significantly.

**TABLE 1:** Means, Standard Deviations, ANOVA of Differences at Pretreatment, and Analyses of Covariance of Treatment Outcomes at Posttreatment

	ADHD			Not ADHD			Pre-treatment		Post- treatment		
										*F*	*p*
Variable	Pre Mean (*SD*)	Post Mean (*SD*)	Post Adjusted Mean (*SE*)	Pre Mean (*SD*)	Post Mean (*SD*)	Post Adjusted Mean (*SE*)	*F*	*p*	*df*	For Adjusted Means	
Parent reports											
PDR	25.90 (12.28)	16.37 (11.02)	14.99 (1.20)	23.29 (12.36)	14.62 (11.34)	15.40 (.89)	2.56	.11	196	.07	.79
CBCL Externalizing	26.12 (10.21)	17.99 (10.25)	16.42 (.96)	22.81 (8.71)	16.40 (9.50)	17.26 (.70)	7.42	.01	200	.48	.49
CBCL Internalizing	12.51 (8.25)	9.32 (7.65)	9.51 (.78)	13.12 (7.99)	10.62 (8.39)	10.52 (.57)	.33	.56	200	1.06	.31
CBCL Attention Prob.	9.17 (3.67)	7.59 (3.72)	6.74 (.37)	7.10 (3.42)	5.86 (3.91)	6.33 (.27)	20.15	.00	200	.77	.38
CBCL Social Prob.	5.55 (2.85)	4.10 (2.66)	3.58 (.28)	4.27 (2.96)	3.50 (2.98)	3.79 (.20)	11.35	.00	200	.35	.56
CBCL Anx./Dep.	6.85 (5.01)	5.10 (4.69)	5.10 (.49)	7.22 (4.64)	5.78 (4.80)	5.78 (.35)	.34	.56	200	1.23	.27
SSRS parent	88.21 (12.08)	91.30 (11.74)	92.33 (1.12)	88.99 (11.40)	95.25 (13.17)	94.67 (.83)	.26	.61	214	2.69	.10
Teacher reports											
TRF Externalizing	24.84 (14.20)	21.45 (13.54)	19.57 (1.44)	20.12 (15.10)	18.08 (15.76)	19.17 (1.08)	5.36	.02	180	.05	.83
TRF Internalizing	10.15 (7.35)	9.38 (7.64)	8.81 (.77)	8.89 (6.81)	8.45 (7.34)	8.78 (.57)	1.69	.20	180	.00	.97
TRF Attention Prob.	18.58 (7.14)	16.67 (6.82)	14.99 (.89)	14.22 (8.49)	13.69 (9.55)	14.65 (.66)	15.61	.00	180	.09	.77
TRF Social Prob.	6.37 (4.08)	5.21 (4.22)	4.58 (.43)	4.88 (4.06)	4.72 (4.29)	5.08 (.32)	7.11	.01	180	.84	.36
TRF Anx./Dep.	7.05 (5.63)	6.15 (5.45)	5.69 (.55)	5.86 (4.84)	5.45 (4.72)	5.72 (.41)	2.81	.10	180	.00	.97
SSRS teacher	66.20 (9.43)	68.10 (8.74)	69.89 (1.10)	70.58 (10.98)	71.23 (11.50)	70.20 (.82)	9.36	.00	175	.05	.83
Academic competence	2.73 (.77)	2.80 (.82)	2.90 (.06)	2.98 (.75)	2.95 (.74)	2.89 (.05)	5.51	.02	156	.00	.98

*Note*. Unadjusted pre and post means are reported for all measures. Adjusted means are based on ANCOVA analyses, and adjusted for both pretest scores, type of organization, and gender.

### Clinical Significance

Among the children with CP and ADHD, 36% (*N* = 26 of 97) moved from the clinical to the nonclinical range on parent-reported externalizing problems compared with 30% of children with CP only. Furthermore, among the children with or without co-occurring ADHD, an equal proportion of 16% moved from the clinical range to the nonclinical in teacher ratings of externalizing problems. The percentage remaining in the clinical range on externalizing behavior problems across treatment was the same in both groups (47% in parent ratings), while on teacher ratings, more children remained above the 90th percentile in the combined CP and ADHD group (57%) than in the CP group (45%). The difference was, however, not significant, χ^2^ (1) = .57, *p* = .51.

### Variables Moderating the Association Between ADHD and Outcomes

**FIGURE 1:**
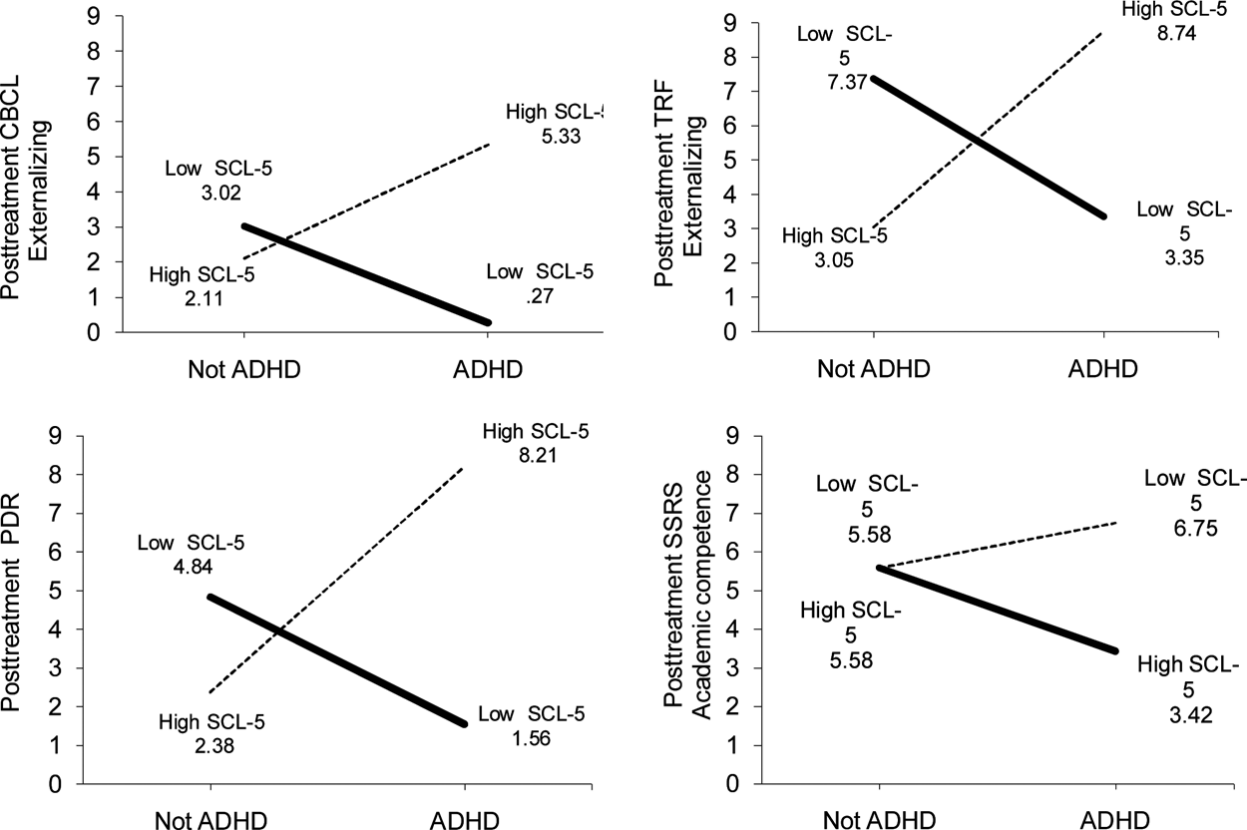
Regression lines for posttreatment scores on parent-reported externalizing (CBCL), teacher-reported externalizing (TRF), parent daily report (PDR), and teacher-reported academic competence (SSRS) as a function of combinations of maternal anxiety/depression and ADHD/not ADHD.

Regression analyses revealed that maternal depression/anxiety (SCL-5) moderated the effects of ADHD on the following outcomes: the PDR (β = .19, *p* < .01, Δ*R* ^2^ = .026), CBCL externalizing behavior (β = .15, *p* < .03, Δ*R* ^2^ = .016), TRF externalizing behavior (β = .15, *p* < .03, Δ*R* ^2^ = .018), and academic competence (β = −.12, *p* < .05, Δ*R* ^2^ = .01). When examining the simple slopes for these interaction effects, ADHD combined with high scores on SCL-5 consistently showed poorer outcomes on PDR (*t*_180_ = 2.18, *p* < .05), parent-reported externalizing behavior (*t*_186_ = 2.05, *p* < .05), academic competence (*t*_145_ = −2.40, *p* < .01), and teacher-reported externalizing behavior (*t*_164_ = 1.65, *p* < .10). By contrast, for children without ADHD, high scores on maternal depression/anxiety were associated with lower scores on teacher-reported externalizing behavior problems (*t*_164_ = −2.01, *p* < .05). For children without ADHD, all other slopes were nonsignificant (Figure 1). These findings indicate that the combination of child ADHD and a high degree of maternal anxiety/depression predicted less beneficial outcomes after PMTO treatment on scores of externalizing problem behavior (TRF, CBCL, and PDR).

**FIGURE 2:**
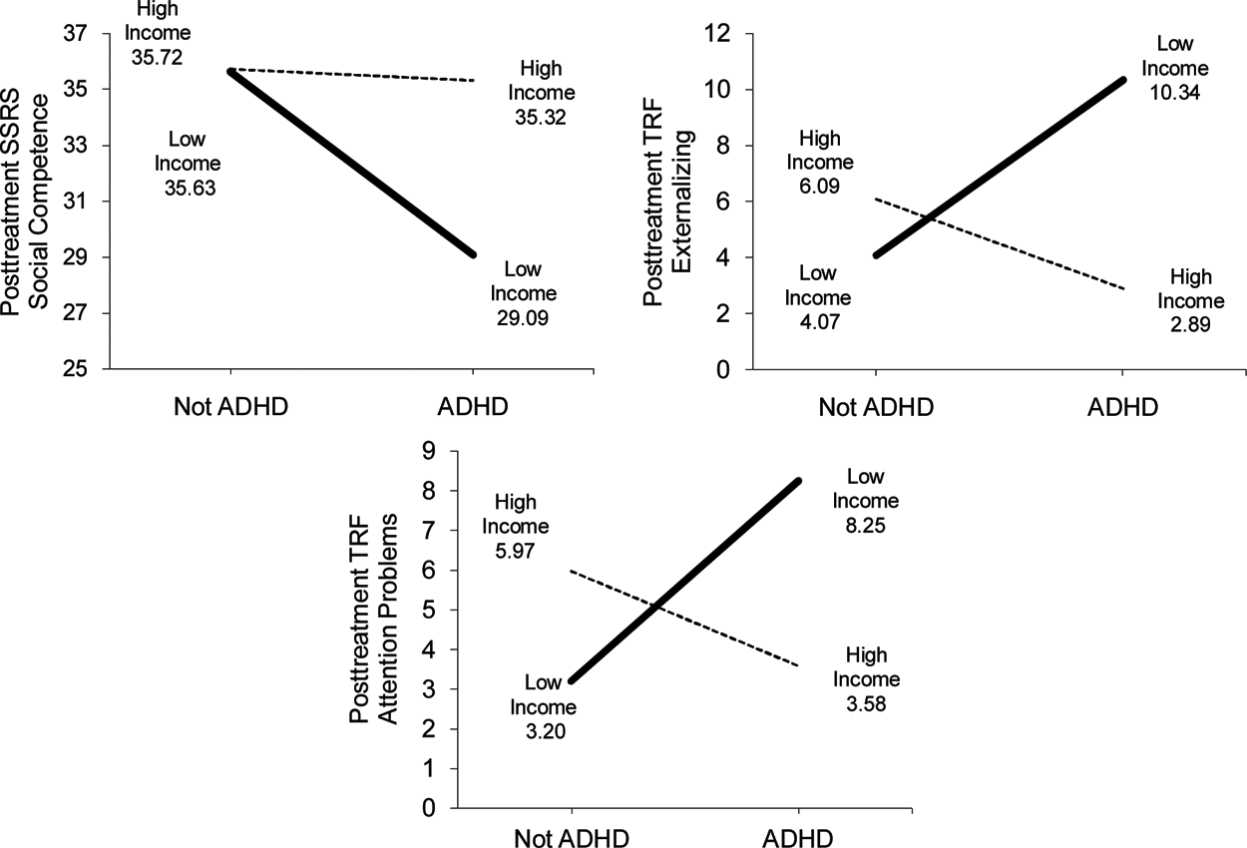
Regression lines for posttreatment scores on teacher-reported externalizing (TRF), parent-reported social competence (SSRS), and teacher-reported attention problems (TRF) as a function of combinations of family income and ADHD/not ADHD.

As illustrated in Figure 2, family income moderated the association between ADHD on the following measures: parent-reported social skills (β = .14, *p* < .03, Δ*R* ^2^ = .013), TRF externalizing behavior (β = −.17, *p* < .01, Δ*R* ^2^ = .02), and TRF attention problems (β = −.23, *p* < .002, Δ*R* ^2^ = .037). The simple slope analyses consistently showed that ADHD combined with low family income predicted poorer outcomes on parent-reported social skills (*t*_207_ = 2.60, *p* < .01), TRF externalizing behavior (*t*_175_ = −2.48, *p* < .01), and TRF attention problems (*t*_175_ = −2.41, *p* < .01). By contrast, for children without reports of an ADHD diagnosis, the simple slope analysis indicated that high family income was associated with poorer outcome on teacher-rated attention problems (*t*_175_ = 2.21, *p* < .05). All other slopes were nonsignificant. Thus, the results indicated that the combination of ADHD and low family income predicted less benefit from PMTO treatment in 3 of 14 analyses.

ADHD combined with single-parent status predicted poorer outcome on parent-reported social skills, *F*(1, 210) = 4.63, *p* < .03, 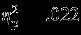. However, only when simple main effects were computed for the children with single parents, children with ADHD were rated lower (*M* = 87.62, *SD* = 8.68) than children without reports of ADHD, *M* = 98.21, *SD* = 12.27, *F*(1, 77) = 13.70, *p* < .01, 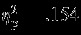. There was no significant difference in social skills ratings between one- and two-parent families for children without an ADHD diagnosis and the differences in social skills rating between single-parent and not single-parent families with children with ADHD was only marginally significant, *F*(1, 74) = 3.42, *p* < .10, 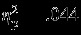. Thus, the findings indicated that children with ADHD who had a single parent, only changed less during treatment than single-parent families with only CP on parent-reported social skills.

Additional analyses were carried out to examine whether the outcomes among children with ADHD were influenced by whether they were reported to be medicated or not (e.g., Ritalin, *n* = 51). In the ANCOVAs, significant predictions of medication appeared for only 1 out of 14 analyses: Children who were medicated had poorer outcomes on teacher-reported attention problems, *F*(1, 65) = 5.44, *p* < .05, 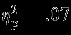; and one marginally significant on teacher-reported social skills, *F*(1, 65) = 3.69, *p* < .06, 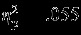.

## DISCUSSION

In this study, in which children with CP with and without co-occurring ADHD were compared, there were no group differences when the social background variables were compared at intake. However, according to both parent and teacher ratings, the children with ADHD had more attention problems, externalizing problems, and social problems than those with CP only. Additionally, teachers reported that children with ADHD had lower academic and social competence. More children with ADHD were recruited from the Child and Adolescent Mental Health Services than from Child Welfare Services. This finding was expected since the primary services in Norway (e.g., school services, child care services, physicians) refer children to these services when they recognize or suspects ADHD. Except for a lower change in parent-rated social skills for children with ADHD, there were no main effects of ADHD on child behavioral outcomes at termination of treatment. Although different at intake, their change in behavioral status did not differ significantly from pretreatment to posttreatment and the two groups did not differ at the end of treatment in terms of their externalized problem behavior and social problems, neither at home nor at school. However, despite a similar magnitude of change, the group with both ADHD and CP still showed higher scores of both teacher- and parent-reported attention problems and social competence at the end for treatment compared to the CP-only group. So, on the one hand, findings indicate that PMTO has produces beneficial change regardless of ADHD. On the other hand, and paradoxically, the higher scores on attention problems and social competence in the group with both ADHD and CP may suggest otherwise.

Examination of interaction effects indicated some interesting findings. First, mothers of children with ADHD who experienced a high degree of depression/anxiety reported significantly poorer outcomes following PMTO on scores on both teacher- and parent-reported externalizing problem behavior. Second, the combination of ADHD and low family income predicted less positive outcomes on parent and teacher ratings of child problem behavior (social competence at home, and externalizing and attention problems at school). Single-parent status did not predict outcomes, except for lower scores on parent-rated social skills. The findings also indicated that medication had a negligible effect on children with ADHD's behavioral status following PMTO, predicting differences in only 1 of 14 outcome variables.

Considering the clinical significance of the outcomes, one third of the children moved from the clinical to the nonclinical range of the externalizing problem behavior according to parent ratings, but only half as many did so according to the teachers. Analyzing movement between the clinical and nonclinical range of externalizing behavior, the children with combined CP and ADHD improved less following PMTO according to teachers than according to parents. However, it should also be mentioned that very few children in the entire group of participants moved from the nonclinical to the clinical range of externalizing problems during treatment; 2% according to parents and 9% according to teachers.

Some limitations of the study should be mentioned. Children were included in the current study based on therapist, parent, and teacher reports of diagnoses of ADHD. Families with missing information about whether the child had been diagnosed or not were excluded from the study. No independent diagnostic evaluation was performed on the participating children. However, the diagnoses were based on earlier assessments conducted by the Child and Adolescent Mental Health agencies in Norway, which follow standardized procedures when assessing children's diagnosis. Moreover, when the diagnoses were validated against the CBCL and TRF attention subscales, it turned out that the group reported to have the diagnosis also scored significantly higher on both parent and teacher ratings of attention problems. These subscales include several items similar to those that define ADHD in the *DSM-IV* manual (American Psychiatric Association [APA], 1994) and in previous studies concepts like ADHD-related behavior (Thapar, Harrington, & McGuffin, 2001) and symptoms of ADHD (Jones, Daley, Hutchings, Bywater, & Eames, 2007) have been used with children with high scores on these types of scales.

Even if behavioral change could be compared across defined subgroups (CP alone and CP + ADHD), this study was unable to demonstrate that the changes were attributable to the intervention given the lack of a comparison group. Furthermore, attrition cannot be dismissed as a potential influence on the findings of the present study, although no significant differences emerged between the attrition group and the completers when compared on background and child behavior/adjustment variables. Behavioral changes following PMTO were analyzed in a pre-post design, and future studies should include follow-up assessments in order to investigate long-term outcomes of PMTO for children with a co-occurring ADHD condition. The current study, carried out in regular services, substantiates the view that co-occuring ADHD did not predict behavioral change in children over and above conduct problems (Beauchine, Webster-Stratton, & Reid, 2005; Hartman et al., 2003; Ollendick et al., 2008).

### Clinical Implications

Even if children with conduct problems, with and without co-occuring ADHD changed equally following parent management training, there were group differences in the combined group indicating that family income and maternal depression predicted less positive child outcomes. It is not clear from these findings if the treatment program should be modified in order to accommodate the challenges represented by these groups, or whether it is sufficient to increase the awareness of therapists working with these children and families in order to increasingly tailor the treatment to these families’ needs.

The results of the study call for efforts to adjust parent training in order to improve outcomes for families with children who responded less well to treatment. As noted, the combination of ADHD and unfavorable contextual factors (e.g., maternal depression/anxiety, low family income) predicted less beneficial child outcomes. Maybe families struggling with multiple problems will respond better if PMTO incorporates a multisystemic and flexible approach when needed. For instance, enhancements can be made to address the particular needs of mentally distressed mothers who receive parent training (Kjøbli et al., 2013). Incorporating modules in PMTO that addresses contextual factors may be of great importance in the continued efforts to improve the lives of children with conduct problems.

## References

[CIT0001] Achenbach T. M. (1991). Integrative guide for the 1991 CBCL/4–18, YSR and TRF profiles.

[CIT0002] Aiken L. S., West S. G. (1991). Multiple regression: Testing and interpreting interactions.

[CIT0003] (1994). Diagnostic and statistical manual of mental disorders.

[CIT0004] Amlund-Hagen K., Ogden T., Bjørnebekk G. (2011). Treatment outcomes and mediators of parent management training: A one-year follow-up of children with conduct problems. Journal of Clinical Child and Adolescent Psychology.

[CIT0005] Askeland E, Christiansen T., Solholm R. (2005). Norwegian handbook of parent management training.

[CIT0006] Beauchine T. P., Webster-Stratton C., Reid M. J. (2005). Mediators, moderators and predictors of 1-year outcomes among children treated for early-onset conduct problems: A latent growth curve analysis. Journal of Consulting and Clinical Psychology.

[CIT0007] Chamberlain P., Reid J. B. (1987). Parent observation and report of child symptoms. Behavioral Assessment.

[CIT0008] Corcoran J., Dattalo P. (2006). Parent involvement in treatment for ADHD: A meta- analysis of the published studies. Research on Social Work Practice.

[CIT0009] Deault L. C. (2010). A systematic review of parenting in relation to the development of comorbidities and functional impairments in children with attention-deficit/hyperactivity disorder (ADHD). Child Psychiatry and Human Development.

[CIT0010] Fabiano G. A., Pelham W. E., Coles E. K., Gnagy E. M., Chronis-Tuscano A., O'Connor B. C. (2009). A meta-analysis of behavioral treatments for attention-deficit/hyperactivity disorder. Clinical Psychology Review.

[CIT0011] Forgatch M. S. (1994). Parenting through change: A programmed intervention curriculum for groups of single mothers.

[CIT0012] Forgatch M. S., DeGarmo D. S. (1999). Parenting through change: An effective prevention program for single mothers. Journal of Consulting and Clinical Psychology.

[CIT0013] Gittelman R., Mannuzza S., Shenker R., Bonagura N. (1985). Hyperactive boys almost grown up: I. Psychiatric status. Archives of General Psychiatry.

[CIT0014] Gresham F. M., Elliott S. N. (1990). Social Skills Rating System.

[CIT0015] Gresham F. M., Lane K. L., Lambros K. M. (2000). Comorbidity of conduct problems and ADHD: Identification of the “fledging psychopaths.”. Journal of Emotional and Behavioral Disorders.

[CIT0016] Hartman R. R., Stage S., Webster-Stratton C. (2003). A growth curve analysis of parent training outcomes: Examining the influence of child risk factors (inattention, impulsivity and hyperactivity problems), parental and family risk factors. Journal of Child Psychology and Psychiatry.

[CIT0017] Hinshaw S. P. (1994). Attention deficits and hyperactivity in children.

[CIT0018] Johnston C., Jassy J. S. (2007). Attention-deficit/hyperactivity disorder and oppositional/conduct problems: Links to parent-child interactions. Journal of the Canadian Academy of Child and Adolescent Psychiatry.

[CIT0019] Jones K., Daley D., Hutchings J., Bywater T., Eames C. (2007). Efficacy of the Incredible Years basic parent training program as an early intervention for children with conduct problems and ADHD. Child Care, Health and Development.

[CIT0020] Kjøbli J., Bjørnebekk G. (2013). A randomized effectiveness trial of brief parent training: Six-month follow-up. Research on Social Work Practice.

[CIT0021] Kjøbli J., Nærde A., Bjørnebekk G., Askeland E. (2013). Maternal mental distress influences child outcomes in brief parent training. Child and Adolescent Mental Health.

[CIT0022] Lee P., Niew W., Yang H., Chen V. C., Lin K. (2012). A meta-analysis of behavioral parent training for children with attention deficit hyperactivity disorder. Research in Developmental Disabilities.

[CIT0023] Lundahl B., Risser H. J., Lovejoy M. C. (2006). A meta-analysis of parent training: Moderators and follow-up effects. Clinical Psychology Review.

[CIT0024] Lurie J. (2006). Teachers’ perceptions of emotional and behavioral problems in 6–12 year old Norwegian school children.

[CIT0025] Lynam D. R. (1998). Early identification of the fledgling psychopath: Locating the psychopathic child in the current nomenclature. Journal of Abnormal Psychology.

[CIT0026] Manuzza S., Klein R. G., Abikoff H., Moulton J. L. (2004). Significance of childhood conduct problems to later development of conduct disorder among children with ADHD: A prospective follow-up study. Journal of Abnormal Child Psychology.

[CIT0027] Moffitt T. E. (1990). Juvenile delinquency and Attention Deficit Disorder: Boys’ developmental trajectories from age 3 to age 15. Child Development.

[CIT0028] Nøvik T. S. (1999).

[CIT0029] Ogden T. (2003). The validity of teacher ratings of adolescents’ social skills. Scandinavian Journal of Educational Research.

[CIT0030] Ogden T., Amlund-Hagen K. (2008). Treatment effectiveness of parent management training in Norway: A randomized controlled trial of children with conduct problems. Journal of Consulting and Clinical Psychology.

[CIT0031] Ogden T., Forgatch M., Askeland E., Patterson G. R., Bullock B. (2005). Implementation of parent management training at the national level: The case of Norway. Journal of Social Work Practice.

[CIT0032] Ollendick T. H., Jarrett M. A., Grills-Taquechel A., Hovey L. D., Wolff J. C. (2008). Comorbidity as a predictor and moderator of treatment outcome in youth with anxiety, affective, attention deficit/hyperactivity disorder, and oppositional/conduct disorders. Clinical Psychology Review.

[CIT0033] Owens E. B., Hinshaw S. P., Kraemer H. C., Arnold L. E., Abikoff H. B., Cantwell D. P., Wigal T. (2003). Which treatment for whom for ADHD? Moderators of treatment response in the MTA. Journal of Consulting and Clinical Psychology.

[CIT0034] Patterson G. R. (1986). Performance models for antisocial boys. American Psychologist.

[CIT0035] Patterson G. R., Chamberlain P., Reid J. B. (1982). A comparative evaluation of a parent-training program. Behavior Therapy.

[CIT0036] Patterson G. R., Forgatch M. S. (2005). Parents and adolescents living together: Part 1: The basics.

[CIT0037] Preacher K. J., Curran P. J., Bauer D. J. (2006). Computational tools for probing interaction effects in multiple linear regression, multilevel modeling, and latent curve analysis. Journal of Educational and Behavioral Statistics.

[CIT0038] Reyno R. M., McGrath P. J. (2006). Predictors of parent training efficacy for child externalizing behavior problems—A meta-analytic review. Journal of Child Psychology and Psychiatry.

[CIT0039] Scott S., Knapp M., Henderson J., Maughan B. (2001). Multicentre controlled trial of parenting groups for childhood antisocial behaviour in clinical practice. British Medical Journal.

[CIT0040] Shelleby E. C., Kolko D. J. (2013). Predictors, moderators, and treatment parameters of community and clinic-based treatment for child disruptive behavior disorders. Journal of Child and Family Studies.

[CIT0041] Tabachnick B. G., Fidell L. S. (2001). Using multivariate statistics.

[CIT0042] Tambs K., Moum T. (1993). How well can a few questionnaire items indicate anxiety and depression?. Acta Psychiatrica Scandinavica.

[CIT0043] Thapar A., Harrington R., McGuffin P. (2001). Examining the comorbidity of ADHD-related behaviours and conduct problems using a twin study design. British Journal of Psychiatry.

[CIT0044] Walker J., Lahey B., Hynd G., Frame C. (1987). Comparison of specific patterns of antisocial behavior in children with conduct disorder with or without coexisting hyperactivity. Journal of Consulting and Clinical Psychology.

[CIT0045] Webster-Stratton C., Lindsay D. W. (1999). Social competence and early-onset conduct problems: Issues in assessment. Journal of Child Clinical Psychology.

[CIT0046] Yoshimasu K., Barbaresi W. J., Colligan R. C., Voigt R. G., Killian J. M., Weaver A. L., Katusic S. K. (2012). Childhood ADHD is strongly associated with a broad range of psychiatric disorders during adolescence: A population-based birth cohort study. Journal of Child Psychology and Psychiatry.

[CIT0047] Zachor D. A., Hodgens J. B., Patterson C. S., Matson J. L., Adrasik F., Matson M. L. (2009). Treatment of attention-deficit hyperactivity disorder (ADHD). Treating childhood psychopathology and developmental disabilities.

